# A Model for Dry Electron Beam Etching of Resist

**DOI:** 10.3390/polym16202880

**Published:** 2024-10-12

**Authors:** Fedor Sidorov, Alexander Rogozhin

**Affiliations:** Valiev Institute of Physics and Technology of Russian Academy of Sciences, Moscow 117218, Russia

**Keywords:** PMMA, e-beam lithography, thermal depolymerization, thermal reflow

## Abstract

This paper presents a detailed physical model for a novel method of two- and three-dimensional microstructure formation: dry electron beam etching of the resist (DEBER). This method is based on the electron-beam induced thermal depolymerization of positive resist, and its advantages include high throughput and relative simplicity compared to other microstructuring techniques. However, the exact mechanism of profile formation in DEBER has been unclear until now, hindering the optimization of this technique for certain applications. The developed model takes into account the major DEBER phenomena: e-beam scattering in resist and substrate, e-beam induced main-chain scissions of resist molecules, thermal depolymerization of resist, monomer diffusion, and resist reflow. Based on the developed model, a simulation algorithm was implemented, which allowed simulation of the profile obtained in resist by DEBER. Experimental verification of the DEBER model was carried out, which demonstrated the reliability of the model and its applicability for theoretical study of this method. The ultimate DEBER characteristics were estimated by simulation. The minimum line width and the maximum profile slope that could be obtained by DEBER were approximately 300 nm and 70°, respectively.

## 1. Introduction

Fabrication of two- and three-dimensional microstructures is a crucial process in various fields such as microelectronics, microengineering, diffraction optics, and nanophotonics [1,2,3,4,5,6]. A number of microstructuring methods exist, but for any particular method, advantages such as versatility, high throughput, and availability are often mutually exclusive. Universal methods with high resolution (grayscale e-beam lithography, two-photon laser lithography, and scanning probe lithography) require highly precise systems and have extremely low throughput. On the other hand, more affordable and productive methods (interference lithography, nanoimprint lithography) allow only structures of a certain type to be obtained.

**Figure 1:**
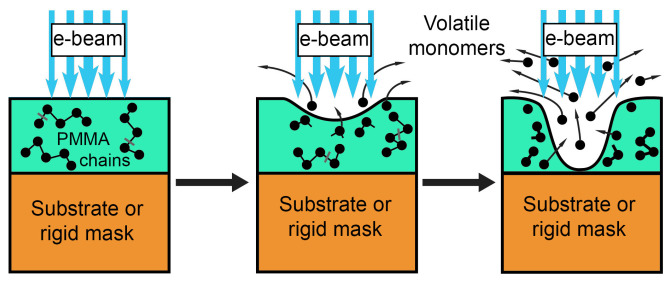
The scheme of dry e-beam etching of resist (DEBER).

In view of the above, attention should be paid to the novel one-stage lithographic method dry electron beam etching of resist (DEBER). This method is based on a chain reaction of thermal depolymerization, which takes place in positive polymer resists during e-beam exposure at temperatures above the glass transition. This reaction results in the relief formation directly during the exposure [7,8] (Figure 1). The features of DEBER include extremely high resist sensitivity, high vertical resolution, the ability to form three-dimensional structural formations without a development stage, and the well-rounded profile of the obtained structures. High resist sensitivity in DEBER significantly increases the throughput of this method compared to standard “wet” e-beam lithography. Due to these features DEBER can be used for the formation of various microelectromechanical systems, optoelectronic devices, diffraction and holographic optical elements, masks, and other three-dimensional microstructures.

**Figure 2:**
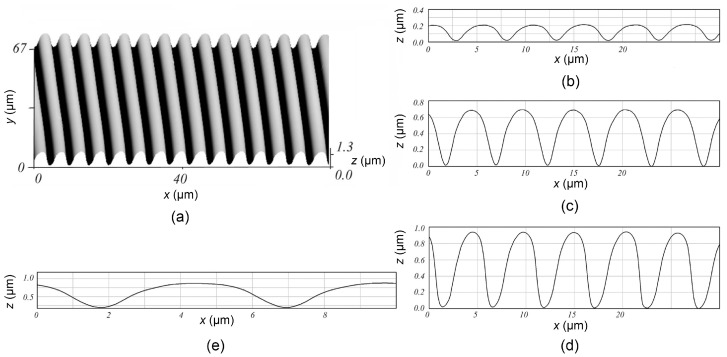
The gratings obtained by DEBER in a PMMA layer with an initial thickness of 900 nm by the exposing it in a series of parallel lines at 160 °C: (**a**) 3D image; (**b**–**d**) profiles, obtained with exposure doses of 0.05, 0.2 and 0.87 μC/cm^2^, respectively; (**e**) profile (**d**) at a scale of 1:1 [8].

The most recent results of DEBER experimental studies are provided in the papers [8,9]. It was demonstrated that the profiles obtained by exposure in a series of parallel lines (“in frame” exposure) have an almost sinusoidal shape (Figure 2), which supports using DEBER for the formation of diffraction and holographic optical elements. The high throughput of the method has also been noted; at a temperature of 160 °C, total etching at the line center was achieved with an exposure dose of less than 1 μC/cm^2^. In addition, the profiles obtained in the PMMA layer by DEBER were transferred to tungsten and silicon through etching in an inductively coupled plasma.

The advantages of DEBER include high throughput and relative simplicity. This enables DEBER to be implemented in a wide range of electron beam systems with minor modifications. Additionally, the specific well-rounded profile shape caused by resist thermal reflow may be beneficial for certain applications [4]. The main disadvantages of DEBER are the low lateral resolution and low aspect ratio of obtained structures. The use of electron beam systems with a beam diameter around 10 nm allows us to obtain the structures with a minimum line width of 300–400 nm (at half-depth) and a maximum profile slope of approximately 20° [8]. These characteristics could be improved by using modern electron beam lithography systems with modifications that allow sample heating and effective removal of volatile monomers. Along with this, few organizations are willing to provide these expensive facilities for an under-researched method, let alone making modifications and dealing with the risk of monomer contamination. Thus, for the further development of DEBER, a theoretical study of this method is required in order to identify its applicability limits and optimize it for specific fields.

## 2. Theory and Methods

**Figure 3:**
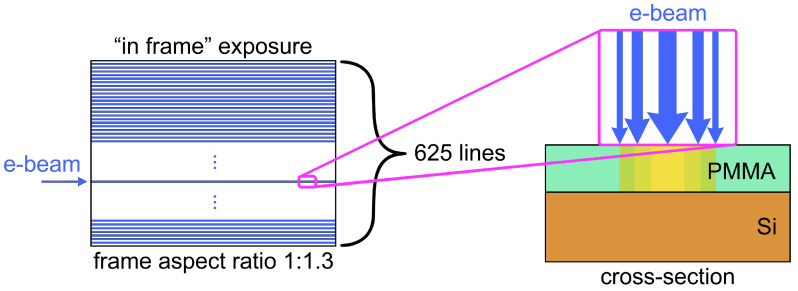
The depiction of “in frame” exposure schema (exposure in series of parallel lines).

The major phenomena of DEBER are electron beam scattering in the resist and substrate, e-beam-induced resist main-chain scissions, e-beam-induced resist thermal depolymerization, monomer diffusion, and resist thermal reflow. In this section, firstly, the individual approaches to simulation of DEBER phenomena will be presented. Then, the model for the overall DEBER process and the simulation algorithm for the profile obtained by DEBER will be discussed. Based on previous experiments, the development of the DEBER model will be carried out for the case of PMMA/Si samples. Also, “in frame” exposure (Figure 3), which was typical for most experiments, is taken into account.

### 2.1. E-Beam Scattering in the PMMA/Si System

For the simulation of electron beam scattering in the PMMA/Si sample, a Monte Carlo algorithm was implemented. Elastic scattering was described in terms of Mott differential cross sections:

(1) $$\frac{d\sigma_{el}}{d\Omega }={\left|f\left(\theta \right)\right|}^{2}+{\left|g\left(\theta \right)\right|}^{2},$$

where f(θ) and g(θ) represent scattering amplitudes, corresponding to parallel and antiparallel electron spin directions [10].

To describe inelastic scattering in PMMA, a model considering electron-electron, electron-phonon and electron-polaron scattering [11] was applied. In turn, inelastic scattering in Si was described using the approach based on Drude oscillators [12]. A Monte Carlo simulation of electron beam scattering in a PMMA/Si sample was used to obtain the distribution of electron-electron scattering events in PMMA, which was then used for the simulation of electron beam induced PMMA main-chain scissions.

### 2.2. E-Beam Induced PMMA Main-Chain Scissions

The quantitative characteristic of radiation-induced polymer main-chain scissions is the radiation scission yield ($G_{s}$): the number of main-chain scission, corresponding to the energy deposition of 100 eV. $G_{s}$ can be determined experimentally by measuring the number average molecular weight of the PMMA sample before and after exposure ($M_{n}$ and $M_{n}^{\prime }$, respectively [13]):

(2) $$M_{n}^{\prime }=\frac{M_{n}}{1+\frac{G_{s}\varepsilon }{100\rho N_{A}}},$$

where ε is energy deposition density, ρ is polymer density, $N_{A}$ is Avogadro’s number, and the number 100 denotes the molecular weight of PMMA monomer. According to Charlesby, in case of e-beam irradiation of PMMA at room temperature, the value of $G_{s}$ is approximately 1.8. $G_{s}$ also increases with temperature, showing a nearly linear relationship between $\ln(G_{s})$ and 1/T [14].

According to Stepanova, PMMA main-chain scissions are caused by incident electron interaction with C–C bonds in the PMMA main-chain [15,16]. Thus, to simulate e-beam induced PMMA main-chain scissions at higher temperatures, a model was developed based on the assumption that an electron-electron scattering event results in PMMA main-chain scission with a certain probability $p_{s}$. In this case, the experimentally observed $G_{s}$ increase at higher temperatures can be explained by an increasing the $p_{s}$. To determine the dependence of $p_{s}$ on temperature, a simulation of the experiment was carried out, in which $G_{s}$ was determined from PMMA number average molecular weight before and after exposure. The parameters of the experiment were taken from the work of Harris [17]: PMMA layer thickness, 500 nm; Si substrate, electron beam energy, 10 keV; exposure dose, 100 μC/cm^2^. The initial values of PMMA number average and mass average molecular weight were 563,000 and 2,260,000, respectively.

**Figure 4:**
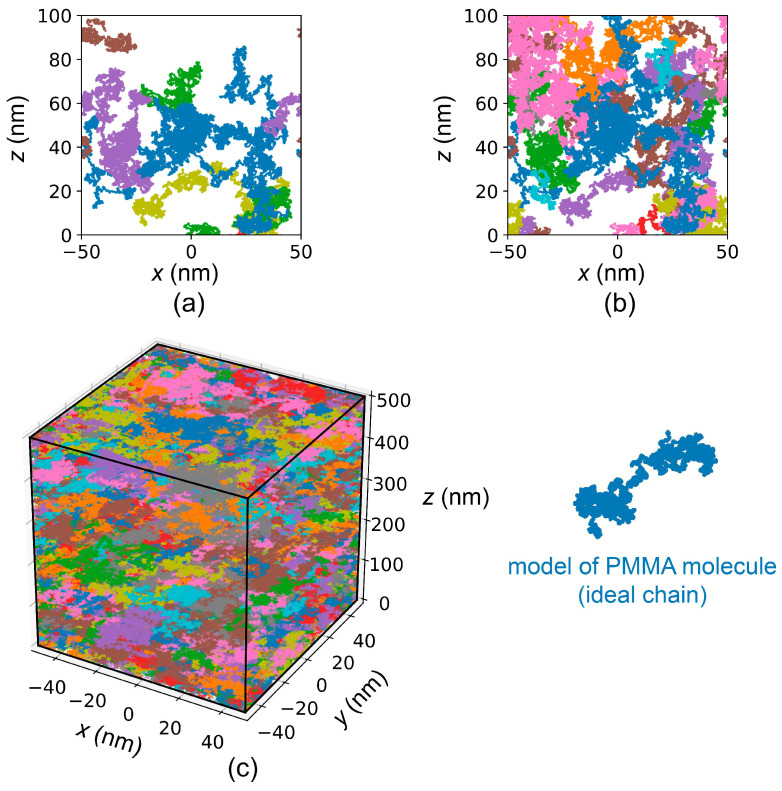
Illustration of the process of filling the volume with models of PMMA molecules (**a**,**b**) while forming the model of the PMMA layer (**c**).

To simulate the number average molecular weight of exposed PMMA, a model for PMMA layer was developed which provided detailed information about the molecular mass distribution. Initially, individual molecules were simulated using an ideal chain model [18]. The molecule lengths were consistent with the initial PMMA molecular weight distribution [17]. The position of each molecule was randomly selected within a 100×100×500 nm^3^ volume, taking into account periodic boundary conditions. This volume was filled with molecule models until the desired density was reached (1.19 g/cm^3^) (Figure 4). Then, e-beam exposure of PMMA/Si sample was simulated under the assumption of a uniform dose distribution over a 100 × 100 nm^2^ area with periodic boundary conditions. After the simulation of exposure, the volume was divided into 5 × 5 × 5 nm^3^ cells. For each cell the corresponding number of simulated electron-electron scattering events ($N_{e-e}\left[i,j,k\right]$) was determined. For each simulated electron-electron scattering event it was determined whether it led to a PMMA main-chain scission:

$$\begin{array}{cc} \xi <p_{s} & \Rightarrow \mathrm{scission},\\ \xi \geq p_{s} & \Rightarrow \mathrm{no}\;\mathrm{scission}, \end{array}$$

where ξ is a random number drawn from uniform distribution over [0, 1) interval. Thus, by setting $p_{s}$, one can simulate the number of PMMA main-chain scissions in each cell of the PMMA layer ($N_{s}\left[i,j,k\right]$).

For each cell of the PMMA layer, the molecules and monomers that belonged to it were identified. Then, $N_{s}\left[i,j,k\right]$ monomers were randomly chosen and assigned the scissions in this cell. The numbers of molecules passing through each cell and monomer positions in each molecule were stored in computer memory, so this approach allowed simulation of the scission positions in each molecule in the model of the PMMA layer. With known scission positions in each molecule, the number average molecular weight of the exposed PMMA was determined directly, and the experimental value of $G_{s}$ was simulated using Formula (2).

The model described above required calibration, necessary to determine the dependence of $p_{s}$ on temperature. To do this, experimental values of $G_{s}$, given in the paper [14], were approximated using the function

(3) $$\ln\left(G_{s}\right)=\frac{k}{T}+b.$$

As a result of this approximation, values of −454.01 for *k* and 2.01 for *b* were obtained. Then, for temperatures relevant to DEBER (120–170 °C), $G_{s}$ values were calculated using Formula (3). Finally, for each temperature, the value of $p_{s}$ that produced the same $G_{s}$ value was determined. Table 1 shows $p_{s}$ values calculated for the temperatures 130, 140, and 150 °C in five independent simulations. The difference between $p_{s}$ values for each temperature in different simulations was less than 1%, so it was decided to ignore the statistical error in $p_{s}$. Thereafter, $p_{s}$ values averaged over the five independent simulations were used in the DEBER model.

**Figure 5:**
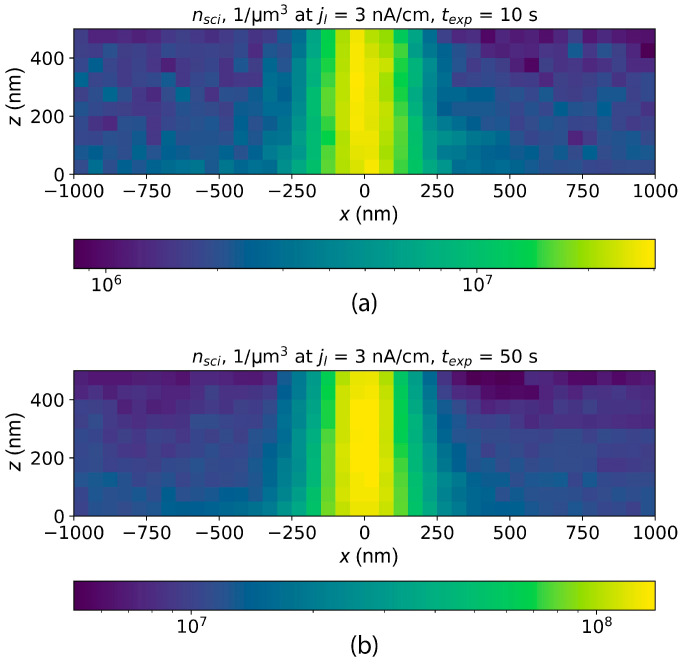
Simulated distributions of PMMA main-chain scission concentration for exposure at a temperature of 130 °C. The exposure is carried out “in frame”; frame dimensions are 2.4 × 1.9 mm^2^, the number of lines in frame is 625, line pitch is 3 μm, exposure time is 10 s (**a**) and 50 s (**b**). Electron beam energy is 20 keV, exposure current is 5 nA.

The simulated distributions of PMMA main-chain scission concentration for two different exposure times are shown in Figure 5.

### 2.3. PMMA Thermal Depolymerization

Electron-beam exposure of PMMA, carried out at higher temperatures, initiates PMMA thermal depolymerization, the process of subsequent detachment of monomers from PMMA molecules. This process leads to the formation of a large amount of free monomers in the PMMA layer and changes the molecular weight distribution of PMMA.

This section presents the approach developed for the simulation of local number average molecular weight of PMMA after exposure at higher temperatures. This approach is based on the model proposed by Boyd, which takes into account the generation of the depolymerization active center due to main-chain scission at a random point inside the polymer molecule: initiation of depolymerization kinetic chain, kinetic chain growth, and kinetic chain termination [19]. In terms of molecular weight distribution moments, the model is described by a set of equations:

(4) $$\frac{dM_{i}}{dt}=k_{S}\left(\frac{2}{i+1}-1\right)M_{i+1}+\frac{dM_{0}}{dt}-k_{S}M_{1}-\frac{i}{\gamma }\left(k_{S}M_{i}+\frac{dM_{i-1}}{dt}\right)\;\left(i\geq 1\right),$$

where $k_{s}$ is the rate constant of active center generation, or the number of active depolymerization centers produced within 1 second per monomer, 1/γ represents the average kinetic chain length during PMMA depolymerization, or the average number of free monomers produced by each active depolymerization center, and $M_{i}$ is the moment of *i*-th order of the polymer molecular weight distribution ($P_{n}$ is a number of molecules with polymerization degree of *n*):

(5) $$M_{i}=\sum_{n=2}^{\infty }n^{i}P_{n}.$$

According to Boyd, the application of this model could be significantly simplified in the case of a Schulz–Zimm polymer molecular weight distribution [20]:

(6) $$P_{n}=C_{0}n^{z}\exp\left(-n/y\right),$$

where $P_{n}$ is the total number of molecules with polymerization degree of *n*, and $C_{0}$ is a normalization factor. The parameter *z* characterizes the width of molecular weight distribution:

(7) $$M_{w}/M_{n}=\left(z+2\right)/\left(z+1\right),$$

where $M_{n}$ and $M_{w}$ are the number average and weight average molecular weight, respectively, and parameter *y* is calculated using the formula

(8) x=y(z+1),

where *x* is the number average polymerization degree. In the case of the Schulz–Zimm distribution function, higher moments can be calculated using the parameters *y* and *z* and the first order moment ($M_{1}$):

(9) $$M_{i}=M_{1}\prod_{n=2}^{i}\left(z+n\right)y^{i-1}.$$

This reduces the number of equations required to 3 for the functions $M_{1}\left(t\right)$, y(t) and z(t).

**Figure 6:**
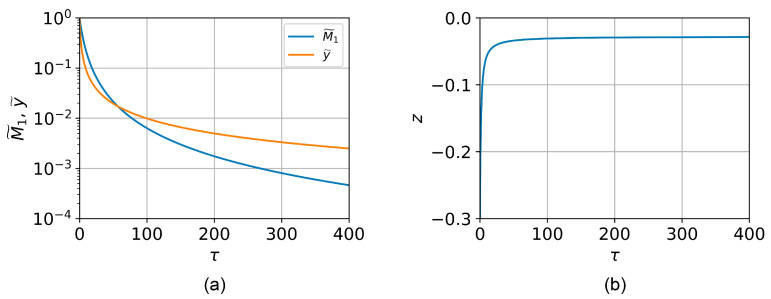
The dependencies of Schultz–Zimm distribution parameters $\tilde{M_{1}}$, $\tilde{y}$ (**a**), and *z* (**b**) on the dimensionless variable τ.

This model was used to simulate the changes in PMMA molecular weight distribution in DEBER. The assumption was made that PMMA molecular weight distribution followed a Schultz–Zimm distribution function with number average and weight average molecular weight values of 271,000 and 669,000, respectively. These values correspond to PMMA 950K A2 (“Allresist”) resist, which was used in this study as well as in the previous experiments. The initial values for the parameters of the Schultz–Zimm distribution ($y_{0}$ and $z_{0}$) were determined using the following equations:

(10) $$\begin{array}{cc} \; & x_{0}=\frac{M_{n0}}{M_{MMA}}=\frac{271,000}{100}=2710,\\ \; & \frac{M_{w0}}{M_{n0}}=\frac{669,000}{271,000}≅2.47,\\ \; & z_{0}=\left(2-\frac{M_{w0}}{M_{n0}}\right)/\left(\frac{M_{w0}}{M_{n0}}-1\right)≅-0.32,\\ \; & y_{0}=x_{0}/\left(z_{0}+1\right)≅3989, \end{array}$$

where $M_{MMA}$ is the molecular weight of the PMMA monomer (methyl methacrylate, MMA). Additionally, the ratios of depolymerization and termination rate constants provided by Mita [21] were used to estimate the average kinetic chain length during PMMA thermal depolymerization in DEBER. After setting all the parameters, the set of differential equations was solved numerically using the following dimensionless variables (Figure 6):

(11) $$\begin{array}{cc} \; & \tau =y_{0}k_{S}t,\\ \; & {\tilde{M}}_{1}=M_{1}/M_{10},\\ \; & \tilde{y}=y/y_{0},\\ \; & \tilde{\gamma }=\gamma y_{0},\\ \; & \tilde{x}=x/x_{0}=\frac{y(z+1)}{y_{0}\left(z_{0}+1\right)}, \end{array}$$

where subscript “0” denotes the initial value. The solutions to the equations allowed us to obtain the dependencies of the PMMA 950K A2 molecular weight distribution parameters on τ.

In order to reduce the computing time, a simulaiton of PMMA local $M_{n}$ was carried out for a line segment with a length of 100 nm along the *y*-axis. The contributions of the other line segments and the other lines were taken into account using periodic boundary conditions. The simulation of PMMA local number average molecular weight was carried out in the following way. Firstly, the two-dimensional histogram was created with a bin size of 50 nm along both the *x* and *y* axes to store the local values of τ. To each bin (with indices *i* and *j*) of this histogram, 0 was assigned as the initial τ[i,j] value. After that, for each one-second interval of exposure, the number of PMMA main-chain scissions related to this bin ($N_{sci}^{1s}\left[i,j\right]$) was determined by simulation. It was assumed that each of the PMMA main-chain scissions would result in the formation of an active depolymerization center, so the increase in τ corresponding to 1 s of exposure ($\Delta \tau_{1s}\left[i,j\right]$) was determined by the formula:

(12) $$\Delta \tau_{1s}\left[i,j\right]=y_{0}\times \frac{N_{sci}^{1s}\left[i,j\right]}{1,789,618}\times 1s,$$

where 1,789,618 is the number of monomers in 50 × 100 × 50 nm^3^ volume, calculated based on the PMMA density and MMA molecular weight (1.19 g/cm^3^ and 100 g/mol, respectively). The value of $\Delta \tau_{1s}\left[i,j\right]$ was added to the current value of τ in this bin, and then *y* and *z* values corresponding to the bin were recalculated. This approach allowed us to simulate PMMA local number average molecular weight during the exposure:

(13) $$M_{n}\left[i,j\right]\left(t\right)=y\left[i,j\right]\left(t\right)\cdot \left(z\left[i,j\right]\left(t\right)+1\right).$$

**Figure 7:**
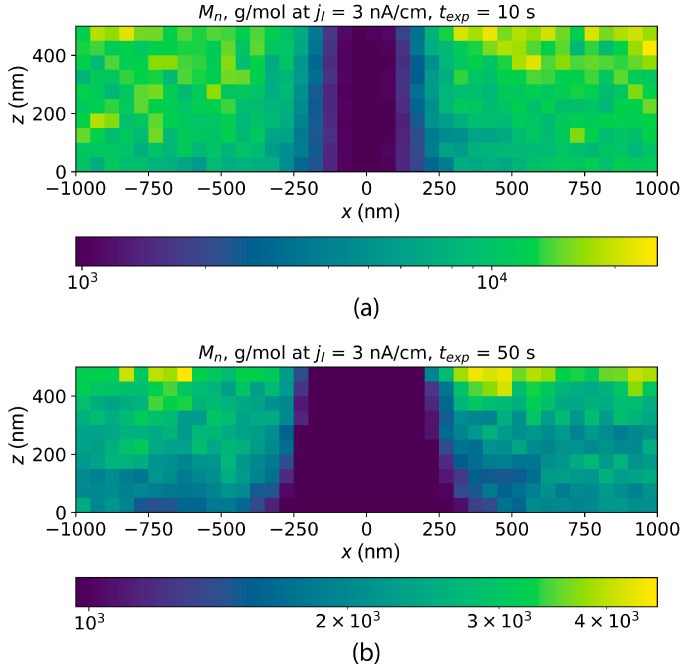
Simulated distributions of PMMA 950K A2 local average molecular weight after exposure at 130 °C. The exposure was carried out “in frame” with a frame size of 2.4 × 1.9 mm^2^, 625 lines per frame, a line pitch of 3 μm, and exposure times of 10 s (**a**) and 50 s (**b**). Electron beam energy was 20 keV, and the exposure current was 5 nA. The simulation was carried out for a 100 nm segment of one line using periodic boundary conditions.

The distributions of PMMA local number average molecular weight simulated for two different exposure times are shown in Figure 7. Taking into account recent experimental studies of DEBER, exposure “in frame” (in a series of parallel lines) was considered. The following parameters were used: line current density, 3 nA/cm; electron beam energy, 20 keV; the exposed area, 2.4 × 1.9 mm^2^; PMMA layer thickness, 500 nm; line pitch, 3 μm; exposure time, 100 s; sample temperature, 130 °C.

### 2.4. Monomer Diffusion in PMMA Layer

As previously mentioned, electron-beam induced PMMA thermal depolymerization results in the formation of significant amount of free monomers in PMMA layer. Once formed, these monomers diffuse within the PMMA layer and out of it. To simulate the diffusion of monomers, the diffusion coefficients for MMA in PMMA provided by Raudino [22] were used. However, the number average molecular weight of PMMA resist used in that study was around 30,000, and for PMMA samples with different $M_{n}$ values the diffusion coefficients may vary. The dependence of diffusion coefficient on $M_{n}$ was taken into account using the empirical equation proposed by Berens [23]:

(14) $$\mathrm{lg}D=\mathrm{lg}D_{\infty }+k/M_{n},$$

where $D_{\infty }$ is the diffusion coefficient corresponding to formally infinite $M_{n}$, $k\approx 1.06\times 10^{4}$.

To estimate the time it takes for monomers to leave the PMMA layer, the lowest of temperatures was considered for which diffusion coefficients were determined in the study of Raudino (135 °C). The initial value of the MMA diffusion coefficient in PMMA at 135 °C ($2\times 10^{-10}$ cm^2^/s) was recalculated for the PMMA 950K A2 resist with a number average molecular weight of 271,000 using the following equations:

(15) $$\left\{\begin{array}{c} \mathrm{lg}D_{30,000}=\mathrm{lg}D_{\infty }+\frac{k}{30,000},\;\\ \mathrm{lg}D_{271,000}=\mathrm{lg}D_{\infty }+\frac{k}{270,000}, \end{array}\right.$$

which resulted in the expression for the initial diffusion coefficient of MMA in PMMA 950K A2 at 135 °C (in cm^2^/s):

(16) $$D_{271,000}^{135{}^{\circ}C}=D_{30,000}\times 10^{k\left(\frac{1}{271,000}-\frac{1}{30,000}\right)}=2\times 10^{-10}\times 10^{1.06\times 10^{4}\left(\frac{1}{271,000}-\frac{1}{30,000}\right)}≅9.7\times 10^{-11}.$$

During the exposure, PMMA local $M_{n}$ decreased, which led to an increase in the diffusion coefficient. Equation (16) can be also used to calculate the diffusion coefficient of MMA in PMMA at temperature of 135 °C for PMMA with a number average molecular weight ($M_{n}$) less than 271,000:

(17) $$D_{M_{n}}^{135{}^{\circ}C}=9.7\times 10^{-11}\times 10^{1.06\times 10^{4}\left(\frac{1}{271,000}-\frac{1}{M_{n}}\right)}\;\;\;(\mathrm{in}\;{\mathrm{cm}}^{2}/s).$$

**Figure 8:**
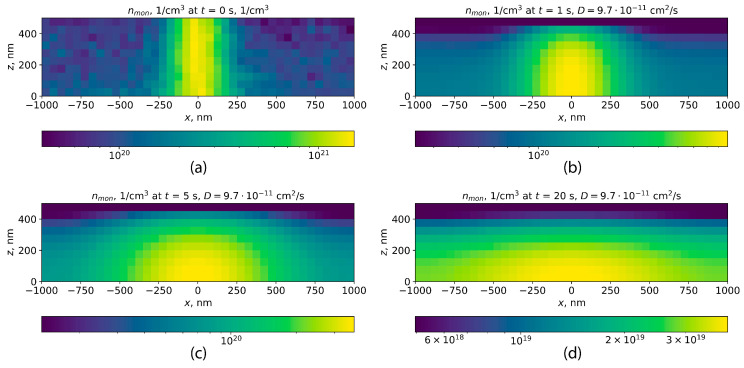
Diffusion simulation for monomers in the case of a diffusion coefficient of $9.7\times 10^{-11}$ cm^2^/s: (**a**) the distribution of initial monomer concentration; (**b**–**d**) the distributions of monomer concentration after 1, 5, and 20 s of monomer diffusion, respectively.

The two-dimensional diffusion equation was numerically solved for the monomers formed in the PMMA layer within one second of exposure in DEBER with the parameters described in the previous subsection. The initial distribution of free monomer concentration in the PMMA layer was determined by multiplying the simulated distribution of PMMA main-chain scissions per one second by the average kinetic chain length during PMMA depolymerization obtained from the study of Mita [21]. Further, the time it takes for monomers to leave the PMMA layer has been estimated as the time required for a tenfold decrease in monomer concentration at the line center. In the case of a diffusion coefficient of $9.7\times 10^{-11}$ cm^2^/s, this time comprises approximately 20 s (Figure 8).

**Figure 9:**
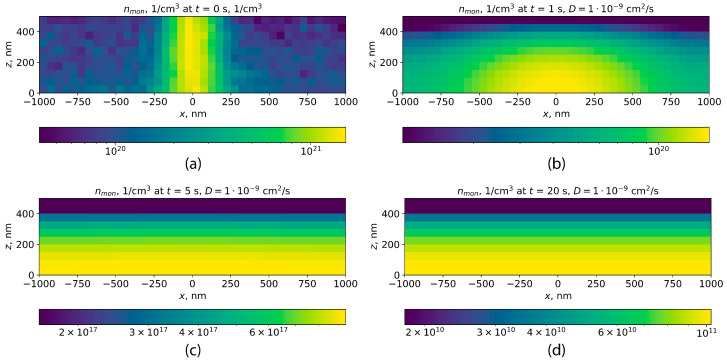
Diffusion simulation for monomers in the case of a diffusion coefficient of $1\times 10^{-9}$ cm^2^/s: (**a**) the distribution of initial monomer concentration; (**b**–**d**) the distributions of monomer concentration after 1, 5, and 20 s of monomer diffusion, respectively.

However, after 10 s of exposure, PMMA number average molecular weight at the line edges decreases to values near $1\times 10^{4}$, which corresponds to diffusion coefficient of approximately $1\times 10^{-9}$ cm^2^/s. In this case, monomer concentration at the line center decreases tenfold within approximately 1 second (Figure 9). Herewith in the line center, where the local $M_{n}$ is lower (Figure 7), the diffusion coefficient will be even higher according to Formula (17). Taking into account the fact that these calculations refer to the lowest of those temperatures at which experimental DEBER studies were carried out, one can be conclude that in DEBER, the time for monomer removal out of the PMMA layer is negligible compared to the typical exposure time. Therefore, it will be further assumed that the free monomers produced by PMMA thermal depolymerization immediately leave the PMMA layer.

### 2.5. PMMA Thermal Reflow

According to previous sections, electron-beam induced PMMA thermal depolymerization results in the intense production of free monomers which leave the PMMA layer almost immediately. Due to this, the PMMA layer becomes more and more sparse, and in this study, such a phenomenon is interpreted as the continuous formation of cavities within the PMMA layer. The volume of these cavities $V_{cav}$ can be calculated by the equation

(18) $$V_{cav}=N_{sci}\cdot 1/\gamma \cdot V_{mon},$$

where $N_{sci}$ is the number of PMMA main-chain scissions, 1/γ is the kinetic chain length during PMMA thermal depolymerization, and $V_{mon}$ is the average monomer volume calculated based on PMMA density and MMA molecular weight ($V_{mon}\approx$ 0.14 nm^3^). Additionally, non-uniform e-beam exposure results in a non-uniform distribution of PMMA number-average molecular weight and, consequently, a non-uniform viscosity profile [24,25]. In our recent study, a mobility-based approach to thermal reflow simulation for PMMA structures with non-uniform viscosity profile was presented [26]. In a nutshell, in this approach, the PMMA viscosity profile is determined using the Williams–Landel–Ferry equation, which describes the dependence of PMMA viscosity (η) on temperature, (*T*) and an empirical formula describing the dependence of PMMA viscosity on $M_{n}$:

(19) $$\begin{array}{cc} \log\left(\frac{\eta (T)}{\eta_{0}\left(T\right)}\right) & =-\frac{C_{1}\left(T-T_{0}\right)}{C_{2}+\left(T-T_{0}\right)},\\ \eta & \propto M_{n}^{\alpha }, \end{array}$$

where $\eta_{0}$, $C_{1}$, $C_{2}$, and $T_{0}$ represent the parameters provided by Aho [27] and Bueche [28]. Then, the free software “Surface Evolver” (version 2.70) [29,30] was used for the finite element simulation of the evolution of PMMA surface during reflow. The non-uniform PMMA viscosity profile was taken into account by the assigning of specific mobility values to the vertices of the PMMA surface. It was determined that for “Surface Evolver” simulation operating in area normalization mode, the vertex mobility μ can be determined from PMMA viscosity η (in Pa·s) by the empirical formula

(20) $$\mu \approx \frac{26.14}{\eta }.$$

**Figure 10:**
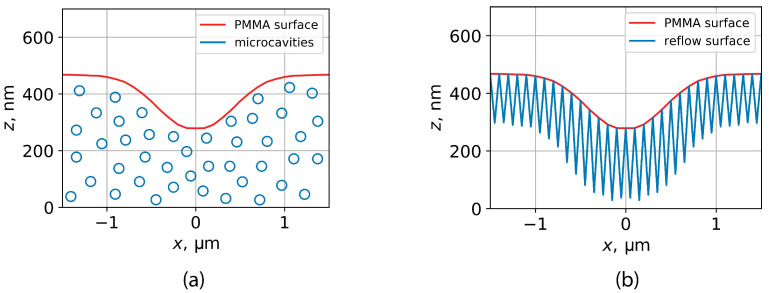
Illustration of an approach to reflow simulation of a sparse PMMA layer (**a**) approximated by a saw-tooth structure (**b**).

The reflow processes in DEBER are caused by surface tension acting on the boundaries of cavities in PMMA layer, and the following approximation was developed to simulate this complex reflow. The PMMA layer was divided in the xy-plane into 100 × 100 nm^2^ segments, and for each segment, the positions and volumes of the cavities were calculated based on a simulated distribution of PMMA main-chain scissions and average kinetic chain length during PMMA depolymerization (Figure 10a). Further, PMMA surface vertices at the middle line of each segment were shifted downward so that the volume of the prism formed under the PMMA surface equaled the total volume of cavities in that segment (Figure 10b). This resulted in a saw-tooth structure that was then reproduced in “Surface Evolver” using the values for vertex mobility calculated from the $M_{n}$ distribution averaged along the *z*-axis. After that, the reflow of the saw-tooth structure was simulated for the required time interval.

This approximation allowed simulation of the complex reflow of a sparse PMMA layer taking into account the non-uniform viscosity profile of PMMA and PMMA volume conservation.

### 2.6. DEBER Model

The combination of the models for DEBER major phenomena described above forms the basis of a model for the overall DEBER process. This allowed the implementation of the simulation algorithm for the profile obtained in PMMA by DEBER. In this algorithm, the total exposure time is divided into one-second intervals, and the following steps are performed for each interval:

1. Simulation of e-beam scattering in PMMA/Si sample;
2. Simulation of e-beam stimulated PMMA main-chain scissions;
3. Simulation of e-beam stimulated PMMA thermal depolymerization;
4. Determination of the mobilities of PMMA surface vertices;
5. Calculation of the positions and volumes of cavities in PMMA layer;
6. Transformation of PMMA layer into a saw-tooth structure;
7. Simulation of thermal reflow of the saw-tooth structure; and
8. Determination of the new positions of PMMA surface vertices.

Finally, the PMMA reflow process during the sample cooling to room temperature was also simulated.

**Figure 11:**
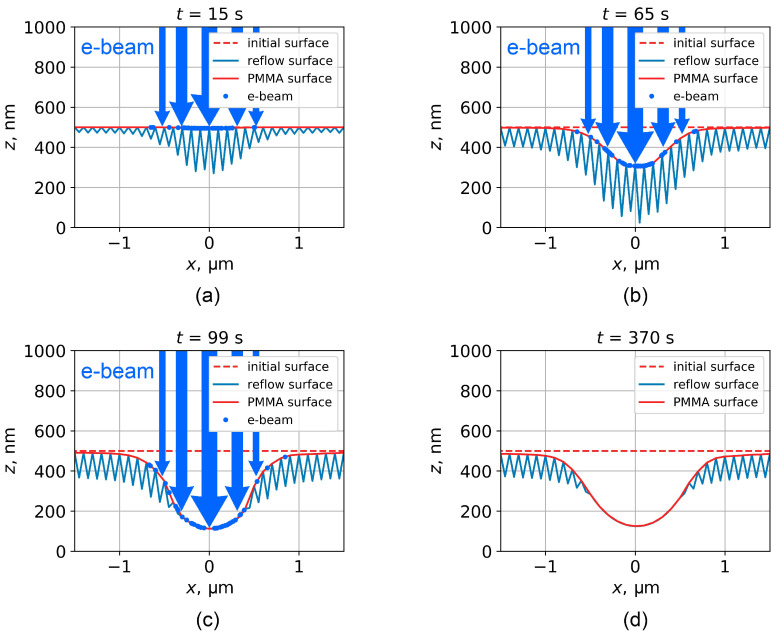
Demonstration of simulation for the profile obtained by DEBER with the following exposure conditions: *E* = 20 keV, *T* = 150 °C/s, exposure line current $j_{l}$ = 20 pA/cm. The exposure is carried out for 100 seconds (**a**–**c**), then the sample cools down at a rate of 1 °C/s until room temperature is reached (**d**). Red dotted line indicates the initial PMMA layer surface, blue dots show the electron entry positions (entry position of every 30th electron is shown). Blue line corresponds to the surface of the saw-tooth structure used to simulate the reflow of a PMMA layer with internal cavities.

The simulated PMMA profiles corresponding to different stages of DEBER process are shown in Figure 11. The simulation was carried out for the following exposure conditions: initial thickness of PMMA layer, 500 nm; electron beam energy *E*, 20 keV; sample temperature *T*, 150 °C/c; exposure time $t_{exp}$, 100 s; exposure line current density $j_{l}$, 20 pA/cm. Current density distribution in the electron beam was assumed to be Gaussian with a standard deviation of 300 nm. After the exposure, the sample was cooled at a rate of 1 °C/s.

Based on the developed model, it is possible to draw a general conclusions about the mechanisms of profile formation in DEBER. At the initial stage of DEBER, e-beam exposure results in PMMA depolymerization and cavity formation in the PMMA layer; however, reflow processes are not yet noticeable due to the relatively high PMMA viscosity (Figure 11a). Then, PMMA number average molecular weight and, consequently, its viscosity decrease, leading to a point where thermal reflow becomes more intense, activating the process of cavity filling (Figure 11b,c). From this point onward, the processes of PMMA depolymerization, new cavity formation, and the filling of existing cavities flow simultaneously with a continuous decrease in PMMA viscosity. At the end of the exposure, these processes fade out, and PMMA reflow during sample cooling is only accompanied by the filling of existing cavities (Figure 11d).

### 2.7. Experiment

**Figure 12:**
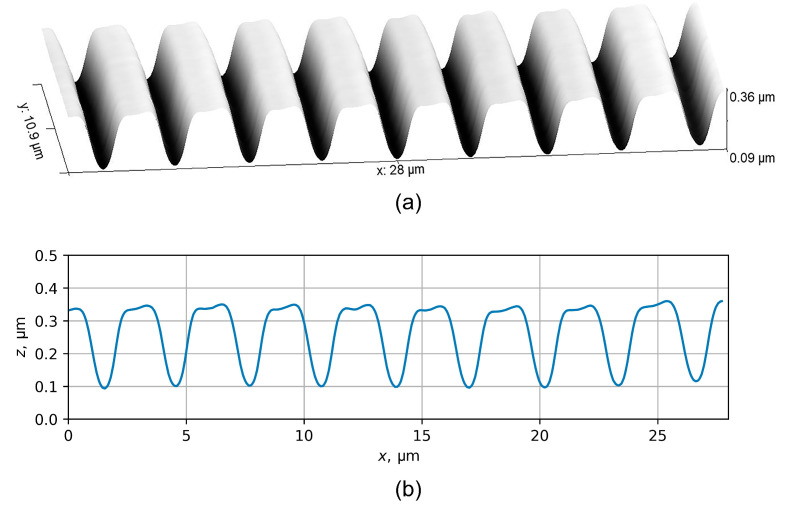
3D image (**a**) and profile (**b**) of the relief obtained in PMMA layer by DEBER under the following conditions: exposure “in frame”, *T* = 150 °C, $t_{exp}$ = 100 s, $D_{l}$ = 3.00 nC/cm. The electron beam energy was 20 keV, and the beam diameter was approximately 600 nm.

**Figure 13:**
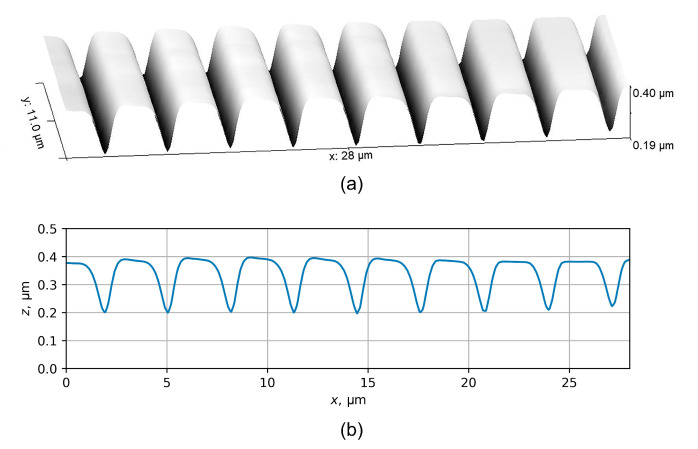
3D image (**a**) and profile (**b**) of the relief obtained in PMMA layer by DEBER under the following conditions: exposure “in frame”, *T* = 130 °C, $t_{exp}$ = 100 s, $D_{l}$ = 3.12 nC/cm. The electron beam energy was 20 keV, and the beam diameter was approximately 600 nm.

The experimental part of this study involved the formation of periodic structures in PMMA/Si samples by DEBER. As in the previous experiments, PMMA 950K A2 (“Allresist”) was used as a resist, and the initial thickness of PMMA layer was 500 nm. The exposure of PMMA/Si samples was carried out using a scanning electron microscope Cambridge Instruments CAMSCAN S4 (Cambridgeshire, UK), which was modified to enable sample heating. The pressure in the microscope chamber was kept at $10^{-5}$ mbar, the electron beam energy was 20 keV, and the beam diameter was approximately 600 nm. The exposure was carried out “in frame”, with a frame size of 2.4 × 1.9 mm^2^ and 625 lines (similar to a standard TV 625-line raster). The exposure current *I* varied in the range of 4.56–5.62 nA and the exposure time $t_{exp}$ varied from 100 to 200 s, so the exposure line dose $D_{l}$ was between 3.00 and 7.38 nC/cm. The sample temperature *T* varied between 130 and 150 °C, and the cooling rate of the substrate after exposure was approximately 0.2 °C/s. The structure profiles were obtained using an atomic force microscope KLA-Tencor Nanopics 2100 (Milpitas, CA, USA). The examples of the profiles obtained in the PMMA layer under the conditions described above are shown in Figure 12 and Figure 13.

## 3. Results

### 3.1. Verification of DEBER Model

For the verification of the developed DEBER model, profile simulation was performed with the exposure parameters corresponding to ones described in the previous section. To reduce the computing time, simulation was carried out for a segment of one line with length of 100 nm. The contribution of other line segments and other lines was taken into account by using periodic boundary conditions. The number of PMMA main-chain scissions, the local average molecular weight of PMMA, and cavity volumes were calculated for cells with dimensions of 100 × 100 × 5 nm^3^ (along the *x*-, *y*-, and *z*-axes, respectively).

**Figure 14:**
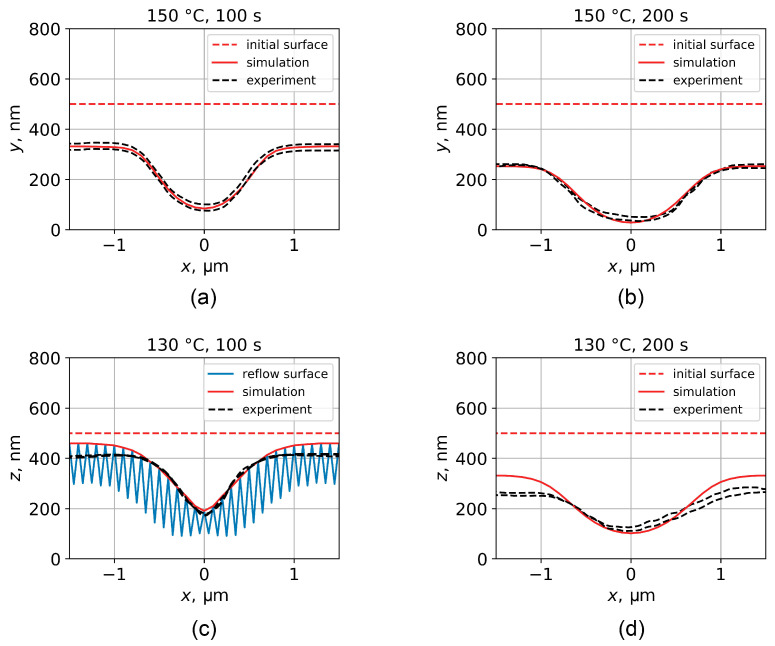
Verification of the developed DEBER model. Comparison of experimental and simulated profiles for the following DEBER conditions: (**a**) *T* = 150 °C, $t_{exp}$ = 100 s, $D_{l}$ = 3.00 nC/cm; (**b**) *T* = 150 °C, $t_{exp}$ = 200 s, $D_{l}$ = 6.73 nC/cm; (**c**) *T* = 130 °C, $t_{exp}$ = 100 s, $D_{l}$ = 3.12 nC/cm; (**d**) *T* = 130 °C, $t_{exp}$ = 200 s, $D_{l}$ = 7.38 nC/cm. In all cases the electron beam energy is 20 keV, and the beam diameter is approximately 600 nm. Black dotted line represents the profiles obtained in the experiment, the red dotted line represents the initial PMMA surface, and the blue line represents the saw-tooth surface that was used to simulate the reflow of the PMMA layer with internal cavities.

A comparison of experimental and simulated profiles is presented in Figure 14. The high level of agreement between the experimental profiles and simulated ones indicates the reliability of the developed model. It was also determined that in case of the exposure conditions described above, the average kinetic chain length during PMMA depolymerization remained constant during the first 100 s of the process, and was equal to 100 and 150 at temperatures 130 and 150 °C, respectively. During the subsequent exposure period (from 100 to 200 s), the average kinetic chain length decreased and its values were 30 and 70 at temperatures of 130 and 150 °C, respectively.

### 3.2. Limits of DEBER Lateral Resolution

The experiments carried out within this study demonstrate that when using an electron beam with a diameter of approximately 600 nm, the profiles obtained by DEBER have a width of around 1 μm at half-depth. The minimum achievable profile width at half-depth can be considered as the ultimate lateral resolution of DEBER and, based on the developed model, two approaches have been identified to improve this value.

Firstly, DEBER lateral resolution can be improved by using a narrow high-energy electron beam. The small beam diameter will help to localize the majority of resist main-chain scissions near the line center, which will lead to intense depolymerization, the generation of free monomers, and a reduction in resist viscosity in this area. Additionally, due to the high energy of the electron beam, the number of scissions far from the line center caused by backscattered electrons will be reduced. In this scenario, it will be necessary to adjust the exposure time in order to ensure that, at the end of the cooling process, the cavities at the line center are filled and those at the line edges remain unfilled. Secondly, the use of a narrow low-energy electron beam can also improve the lateral resolution of DEBER. In the case of low energy of primary electrons, every PMMA main-chain scission will occur near the line center due to relatively low electron penetration depth. This will result in cavities forming only in a small area near the line center, preventing resist from subsiding at the line edges.

**Figure 15:**
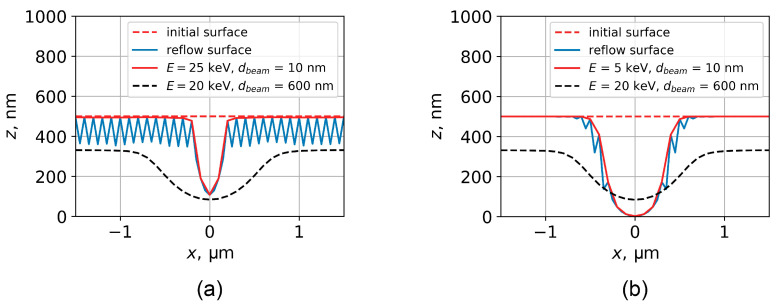
Simulation of the profiles obtained by DEBER using a narrow electron beam (beam diameter is 10 nm) with energy of 25 keV (**a**) and 5 keV (**b**). The sample temperature was 150 °C/s in both cases, and the initial thickness of the PMMA layer was 500 nm. The exposure time was 45 s in both cases. The exposure was carried out along an infinite line; the line current was 30 pA/cm, the cooling rate of the samples was 10 °C/s. For comparison, the black dotted line shows the profile from Figure 14a, obtained in the experiment with a wide beam.

The simulation of profiles obtained by DEBER in the case of using a narrow electron beams with high and low energies is shown in Figure 15. The electron beam diameter was 10 nm, the temperature of the samples was 150 °C/s, and the initial thickness of PMMA layer was 500 nm. The electron beam energy was 25 keV (high-energy beam) and 5 keV (low-energy beam), and the sample cooling rate was 10 °C/s. Exposure was supposed to be carried out along an infinite line. The line current density was 30 pA/cm, and the exposure time was 45 s in both cases.

The simulation showed that the minimum profile width at half-depth can be achieved by using a narrow electron high-energy beam, and that its width was approximately 300 nm. This value was considered to be the ultimate DEBER lateral resolution. Additionally, in the case of using a narrow high-energy beam, the minimum achievable PMMA layer thickness at the line center is limited to around 100 nm due to a relatively large mean free path of electrons at higher energies. On the other hand, using a narrow low-energy electron beam provides a DEBER lateral resolution of only approximately 600 nm; however, it allows total etching of PMMA at the line center due to the shorter mean free path of electrons at lower energies. It should also be noted that the profile obtained by using a narrow low-energy beam is more resistant to reflow processes, as the resist viscosity at the line edges does not decrease significantly. Furthermore, the simulation shows that the maximum achievable profile slope at half-depth for the structures obtained by DEBER was approximately 70° in both cases.

## 4. Discussion and Conclusions

This paper presents a theoretical study of a novel method for the fabrication of three-dimensional microstructures in positive resists, known as dry electron beam etching of resist (DEBER). To date, only experimental studies have been conducted on this method, which has revealed some features of DEBER: high throughput, a smooth relief profile, and limited lateral resolution and low aspect ratio of obtained structures. In most experiments, the resist was exposed “in frame” with a line pitch of several microns, which demonstrated that DEBER can be used to produce the diffraction and holographic optical elements. However, the low lateral resolution and low aspect ratio of the resulting structures limited the applicability of this method. In DEBER, the relief is formed by the combined action of several different processes, and its nature is quite complex. Thus, it has been difficult to identify methods for optimizing DEBER and determining its limits of application based solely on experimental studies.

In DEBER, the major phenomena resulting in profile formation are electron beam scattering in the resist and substrate, e-beam-induced resist main-chain scissions, e-beam-induced resist thermal depolymerization, monomer diffusion, and thermal reflow of resist. These individual processes have been relatively well studied, but their simultaneous flow in the process of profile formation has not been investigated to date. Moreover, it has been found that most of the existing models for DEBER phenomena cannot be used in their original form in order to describe DEBER. For this reason, within this study, new models were developed for e-beam induced PMMA main-chain scissions at higher temperatures and e-beam induced PMMA thermal depolymerization. Additionally, a new approach to PMMA thermal reflow simulation in case of non-uniform viscosity profile was applied. The models of major DEBER phenomena were integrated into a model for the overall DEBER process. Based on this model, a simulation algorithm was implemented for the profile obtained by DEBER with arbitrary process parameters. Experimental verification of the developed DEBER model showed a quite good agreement between experimental and simulated profiles, which enabled the theoretical study of DEBER.

Simulation of DEBER profiles obtained under various exposure conditions provided deep insights into quantitative and qualitative characteristics of DEBER. For example, the significant impact of cavities in the PMMA layer on the line profile was identified. At the end of the cooling stage, the presence of cavities in PMMA layer results in a convex shape to the line edges (Figure 14c), whereas in the absence of cavities, the line edges exhibit a more concave profile (Figure 14a,b,d). In addition, the ultimate DEBER characteristics were estimated, including the minimum line width and maximum profile slope of the structures obtained by this method: 300 nm and 70°, respectively. Moreover, the developed simulation algorithm for the line profile obtained by DEBER can be used to determine DEBER conditions for the formation of the necessary relief. In order to obtain the relief with a profile that matches the ultimate DEBER characteristics, the parameters for exposure and subsequent cooling of the sample can be adjusted through iterative simulation taking into account the features of this method.

## Figures and Tables

**Table 1:** The values of PMMA main-chain scission probability in electron-electron scattering ($p_{s}$) for different temperatures obtained in five independent simulations.

Simulation №	$p_{s}$ at 130 °C	$p_{s}$ at 140 °C	$p_{s}$ at 150 °C
1	8.2844 × 10^−2^	8.5752 × 10^−2^	8.8647 × 10^−2^
2	8.3084 × 10^−2^	8.6012 × 10^−2^	8.8897 × 10^−2^
3	8.2829 × 10^−2^	8.5715 × 10^−2^	8.8526 × 10^−2^
4	8.2834 × 10^−2^	8.5734 × 10^−2^	8.8606 × 10^−2^
5	8.2987 × 10^−2^	8.5880 × 10^−2^	8.8797 × 10^−2^

## Data Availability

The data used to support the findings of this study are included within the article.
